# Stimulated Reporting of Adverse Events Following Immunization with COVID-19 Vaccines

**DOI:** 10.3390/vaccines10122133

**Published:** 2022-12-13

**Authors:** Ratinder Jhaj, Deepa Chaudhary, Ajay K. Shukla, Jayanthi Yadav

**Affiliations:** 1 Madhya Pradesh State AEFI Committee, Coordinator-Regional Training Centre & ADR Monitoring Centre, Department of Pharmacology, All India Institute of Medical Sciences, Bhopal 462020, Madhya Pradesh, India; 2 Regional Training Centre & ADR Monitoring Centre, All India Institute of Medical Sciences, Bhopal 462020, Madhya Pradesh, India; 3 Department of Pharmacology, All India Institute of Medical Sciences, Bhopal 462020, Madhya Pradesh, India; 4 National & Madhya Pradesh State AEFI Committee, Department of Forensic Medicine & Toxicology, All India Institute of Medical Sciences, Bhopal 462020, Madhya Pradesh, India

**Keywords:** Adverse Events Following Immunization, COVID-19, Covishield, Covaxin

## Abstract

In India, up until December 2021, Covishield and Covaxin vaccines against COVID-19 were being used for mass vaccination programs. In view of the urgency of fighting the ongoing pandemic, many vaccines have been granted emergency use approval while phase 2/3 clinical trials were still underway. Even for vaccines that have completed phase 3 trials, safety data may not be comprehensive. This retrospective observational study was conducted at a designated Regional Training Centre for Pharmacovigilance cum Adverse Drug Reaction Monitoring Centre (AMC) under the Pharmacovigilance Programme of India. The data sources were stimulated spontaneous reports of Adverse Events Following Immunization (AEFI) due to the COVID-19 vaccines from 10 January to 31 December 2021. A total of 51,010 COVID vaccine doses were administered during the study period. There were 330 AEFI reported (AEFI rate: 0.65%). Six AEFI were serious events among which three were Adverse Events of Special Interest. The majority of the AEFI were systemic, reported after the first dose, and with an onset between 1 and 24 h after vaccination. On comparing Covishield and Covaxin, there were no statistically significant differences in the AEFI reported with either vaccine in terms of gender, seriousness, lag period, duration, recovery, causality, treatment received for AEFI, presence of co-morbidity, or history of COVID-19 infection. Overall, the rates of AEFI was uncommon, and serious AEFI were rare with both Covishield and Covaxin, with a higher rate after the first dose. Whether immunological tolerance or allayed anxiety was responsible for the lower AEFI risk with the second dose remains to be investigated.

## 1. Introduction

Vaccination has played and continues to play a vital role in stemming the devastating pandemic due to the severe acute respiratory syndrome coronavirus 2 (SARS-CoV-2). As of 22 September 2022, 52 COVID-19 vaccines have been approved fully or for emergency use in different countries while 117 vaccine candidates are in various stages of clinical development [1]. Different strategies are being used and/or developed for vaccines against SARS-CoV-2, including messenger RNA (mRNA) vaccines, virus-vectored vaccines, inactivated virus vaccines, virus-like particle or nanoparticle vaccines, protein subunit vaccines, and DNA and live-attenuated virus vaccines [1]. However, the emergence of variants is a perpetual challenge to the effectiveness of these vaccines. Rapid herd immunity through vaccination is critical to block the mutations and prevent the emergence of variants that can completely escape immune surveillance [2].

In India, up until December 2021, two vaccines against COVID-19 were being used for mass vaccination programs, namely, Covishield and Covaxin. Covishield or the ChAdOx1 nCoV-19 coronavirus vaccine (recombinant) is manufactured by Serum Institute of India Pvt. Ltd. in partnership with AstraZeneca. It is a virus vector-based vaccine that uses replication-deficient chimpanzee adenovirus vector encoding the SARS-CoV-2 Spike (S) glycoprotein, and is produced in genetically modified human embryonic kidney (HEK) 293 cells [3]. Covishield is indicated for the active immunization of individuals of age 18 years or more. It has a two-dose vaccination regimen. Covaxin is India’s indigenous COVID-19 vaccine by developed by Bharat Biotech in collaboration with the Indian Council of Medical Research (ICMR)—National Institute of Virology (NIV) [4]. It is an inactivated virus vaccine developed using whole-virion inactivated vero cell-derived platform technology. Covaxin too has a two-dose vaccination regimen. Both vaccines were approved for emergency use in India on 3 January 2021.

Various adverse effects associated with Covishield and Covaxin have been reported by the manufacturers [3,4,5]. The adverse effects of the above two COVID-19 vaccines reported so far in India have also been listed on the Ministry of Health and Family Welfare (MoHFW) website [6]. A systematic review and meta-analysis of the safety of COVID-19 vaccines in 26 studies revealed an incidence rate of 1.5% (1.4–1.6%) for adverse events, 0.4 (0.2–0.5) per 10, 000 for severe adverse events, and 0.1 (0.1–0.2) per 10,000 for death after vaccination [2]. Four of the studies included AEFI with the ChAdOx1 nCoV-19 vaccine, while there were no studies on Covaxin [2].

However, in view of the urgency of fighting the ongoing pandemic, many vaccines have been granted emergency use approval while phase 2/3 clinical trials were still underway. Even for vaccines that have completed phase 3 trials, safety data may not be comprehensive, as is true for all medicines. This is due to the inherent limitation of randomized clinical trials (RCTs), which usually involve a limited number of homogenous participants for a limited duration of time. Actual, real-world use differs significantly from RCTs in terms of widely heterogeneous populations, vaccine supply, willingness for vaccination, accessibility to vaccines, etc.

It is, therefore, critical to keep a vigilant eye on the emerging adverse effects of the COVID-19 vaccines. We therefore analyzed the Adverse Events Following Immunization (AEFI) reported to the Adverse Drug Reaction (ADR) Monitoring Centre (AMC), during the first year of COVID-19 vaccination, to identify any differences in AEFI reported with the two COVID-19 vaccines (Covishield and Covaxin) administered at our Centre so far, and to evaluate the influence, if any, of factors like age, gender, concomitant drugs or concurrent disease associated with adverse drug events following the two vaccines. The data can also be used to compare the pattern of AEFI with any subsequent vaccine which is used for mass vaccination in the country in future.

## 2. Materials and Methods

Study design: This was a retrospective observational study.

Study Site: The study site is a designated Regional Training Centre for Pharmacovigilance cum Adverse Drug Reaction Monitoring Centre (RTC-AMC) under the Pharmacovigilance Programme of India. All AEFI are also sent via Vigiflow to the Indian Pharmacopeia Commission, Ghaziabad. In addition, serious AEFI are reported to the (State Extended-Programme-On-Immunization Officer (SEPIO) and the District Immunization Officer (DIO). Data used in the study were collected as part of the routine functioning of the ADR monitoring Centre.

Study Sample: All AEFI due to COVID-19 vaccine reported to the Adverse Drug Reaction Monitoring Centre from 10 January 2021 (when COVID vaccination started for HCWs in India) to 31 December 2021 were included. 

Stimulation of Reporting: Contact details of RTC-AMC staff were displayed at the study vaccination center for reporting of AEFI by the COVID-19 vaccine recipients and the vaccination staff. RTC-AMC staff also visited the vaccination site during the vaccination drive to enquire about any AEFI.

Causality assessment: Causality assessment was performed using the WHO Causality Assessment of an adverse event following immunization [7,8].

Statistical Analysis: The adverse drug events reported were analyzed retrospectively for patient characteristics (age, gender, concurrent drug intake, and co-morbidities), type of COVID-19 vaccination (Covishield or Covaxin), vaccine dose (first or second dose of COVID-19 vaccine), previous history of COVID-19 infection, type of adverse event, and its outcome. Adverse events, patient characteristics, and outcomes were expressed as percentages. Chi-square test was used to detect any statistical difference in the frequency and type of AEFI between Covishield and Covaxin. Odds ratio was calculated to identify any association of gender, vaccine dose, or patient category (HCW or non-HCW) with risk of AEFI.

Definitions of AEFI and AESI (Adverse events of Special interest) used were as per the Consultation Document for Case Definitions, Adverse Events of Special Interest, and Adverse Events Following Immunization during COVID-19 Vaccine Introduction, 2022 which provides definitions collected and pooled from different sources, including the Brighton Collaboration (BC) and the WHO, the Safety Platform for Emergency vaccines (SPEAC), and vaccines COVID-19 monitoring readiness (ACCESS) [9].

### Ethical Considerations

The study was approved by the Institutional Human Ethics Committee (IHEC-LOP/2022/IL004 dated 20 February 2022). All the study records were kept confidential, and the identity of the participants was not revealed in any way to unauthorized personnel.

## 3. Results

A total of 51,010 COVID-19 vaccine doses were administered (23,206 first doses and 27,804 second doses) during the study period from 11 January 2021 (the starting date of COVID vaccinations in India) to 31 December 2021. Out of the vaccines administered, 41,462 were Covishield (19,639 first doses and 21,823 second doses) while only 9548 (3567 first doses and 5981 second doses) were Covaxin. Covaxin administration started on 26 April 2021 in the study centre. Of these, 27,633 doses were administered to male recipients while 23,377 were administered to females. Since the data do not include recipient identity, this does not reflect the actual number of men and women who have received the vaccine as one individual received two doses. A total of 6587 (3926 first doses and 2661 second doses) doses were administered to healthcare workers (HCWs), while 44,424 (19,281 first doses and 25,143 second doses) doses were administered to non-HCWs. All patients were above 18 years of age.

During the study period, 330 AEFI (Covishield: 307; Covaxin: 23) were reported, amounting to an AEFI rate of 0.65% (95% CI 0.0058–0.0072) of vaccinations. Most of the AEFI (308 or 93.33%) were reported after the first dose of the vaccine while only 22 (6.67%) were reported after the second dose of the vaccine. Among the 22 patients who had AEFI after the second dose, six had a previous history of AEFI on the first dose, mostly similar symptoms, but none of them had reported the AEFI after their first dose. Males accounted for 188 (56.97%) of the AEFI cases. The mean age of patients reporting AEFI was 38.63 years (40.49 years with Covishield and 36.77 years with Covaxin) A total of 202 AEFI (61.21%) were reported in non-HCWs. Of the remaining number, 61 (18.48%) were in doctors while 67 (20.3%) occurred in other HCWs (nurses, pharmacists, or ancillary staff). (Table 1).

Only six (1.82%) AEFI were classified as serious events — four cases with Covishield and two with Covaxin. Of these, three cases were Adverse Events of Special interest (AESI), that is two cases of anaphylaxis (one each with Covishield and Covaxin) and one case of cerebrovascular accident (CVA) following Covaxin. All serious patients were hospitalized from two hours to three days. The causality for serious AEFI was assessed as probable in five cases and possible in the case of CVA. Five patients recovered fully, while one was recovering at the time of the last follow-up (Table 2).

The majority of the AEFI (270 or 81.82%) were systemic, 4 were localized, while 56 cases presented both systemic and localized symptoms. Pain/tenderness at the site of injection (64 reports) was the most common localized reaction. The most frequent systemic AEFI were fever (244, 27.11%), body ache/stiffness or myalgia (128, 14.22%), and headaches (111, 12.33%). (Table 3 and Table 4).

Most of the AEFI (292 or 88.48%) had an onset between 1 and 24 h after vaccination, while 20 (6.06%) started in less than an hour and only eight (2.42%) AEFI had a lag period of seven days. All AEFI with Covaxin presented within 24 h. (Table 2) The maximum lag period between vaccination and AEFI was 28 days, which included one patient with tingling in the left hand and at the site of injection. Recovery was usually (279 or 84.8%) within 1-5 days, while 12 (3.65%) cases recovered in more than 10 days. Treatment for AEFI was given in 304 (92.12%) cases. (Table 3) Frequently used treatments included nonsteroidal anti-inflammatory medicines (NSAIMs) in 284 (73.2%), proton pump inhibitors and/or antiemetics in 29 (7.47%), and H1 antihistamines in 19 (4.9%) cases.

On causality assessment using the WHO revised classification of AEFI [8], 313 (81.09%) were classified as probable, while 60 (15.54%) were classified as certain, and eight (2.07%) as possible. Five cases (all received Covishield) were thought to be unlikely to be caused by vaccination. All these patients had RT-PCR-confirmed COVID-19 following the Covishield vaccine but were not considered as instances of vaccine failure since only one dose of the vaccine had been received. Moreover, four of these five cases developed symptoms of COVID-19 and tested positive within 1–2 days of vaccination. All had mild cases of the disease not requiring hospitalization. (Table 2).

There was no co-morbidity in 268 (79.53%) patients, while hypertension and diabetes mellitus type 2 were the most common comorbidities at 9.55% and 5.34%, respectively. All but one patient (with CVA) recovered fully. Six patients had a history of COVID-19 infection in the past (all received Covaxin) (Table 1).

We could analyze only three factors, namely, gender, the dose of vaccine (first or second dose), and category of the recipient (HCW or non-HCW) for risk of AEFI following the COVID-19 vaccine, since we had information about only these three factors in those who received the COVID-19 vaccination at the study center but did not report AEFI. We found no association between the risk of experiencing AEFI and gender with either Covishield (OR 1.12; 95% CI 0.89–1.40, *p* = 0.34) or Covaxin (OR 1.00; 95% CI 0.44–2.27, *p* = 1.0). However, non-HCWs had a higher rate of AEFI after receiving Covishield (OR 3.78; 95% CI 3.00–4.75, *p* < 0.0001) as well as Covaxin (OR 9.97; 95% CI 3.36–29.57, *p* < 0.0001) compared to HCWs. What is more, there were a significantly greater number of AEFI reported after the first dose of Covishield (OR 19.23; 95% CI 11.78–31.37, *p* < 0.0001) as well as Covaxin (OR 6.06; 95% CI 2.25–16.34, *p* = 0.0004) when compared to the second dose of either vaccine. (Table 5).

On comparing Covishield and Covaxin, it was found that there were no statistically significant differences in the AEFI reported with either vaccine in terms of gender, seriousness, lag period, duration, recovery, causality, treatment received for AEFI, presence of co-morbidity or history of COVID-19 infection. (Table 5) However, the difference in AEFI after the first dose compared to the second dose was significantly higher after Covishield as compared to Covaxin. Similarly, there were significantly more AEFI in non-HCWs than HCWs after Covishield as compared to Covaxin. Moreover, while there were more vaccine recipients with a co-morbidity (20 or 0.21%) as compared to those with no co-morbidity (5 or 0.05%) in the Covaxin group, the ratio was reversed in the Covishield group with only 64 (or 0.15%) vaccine recipients having a co-morbidity, while the majority (248 or 0.60%) did not have any concurrent disease. (Table 5).

## 4. Discussion

As per a reply by the Government of India in Parliament, up until November 2021, a total of 49,819 AEFI had been reported in India after 123 crore vaccinations (Covishield, Covaxin, and Sputnik), amounting to 0.004% of vaccinations [10]. Of these, a majority, that is, 47,691 were minor, 163 severe, and 1965 serious AEFI. The total deaths and hospitalizations due to COVID vaccines in India were 946 (0.00008%) and 1019 (0.00008%), respectively. However, at our center, we found a higher AEFI rate of 0.65%. Similarly, our proportion of serious AEFI was high at 0.01% of vaccinations or 1.82% of AEFI being serious. The higher AEFI rates (0.65%) found in our study could possibly be attributed to the stimulation of AEFI reporting at the study site by the display of AMC contact details and the visits of RTC-AMC staff at the vaccination center.

Since March 2021, the AstraZeneca vaccine was temporarily suspended in some countries after reports of severe (including several fatal) coagulation disorders [11,12]. Three case series reported unusual thrombotic events combined with thrombocytopenia after vaccination with the ChAdOx1 nCoV19 vaccine. Vaccine-induced prothrombotic immune thrombocytopenia (VIPIT) mainly occurred in women under 55 years of age, prompting many countries to restrict the vaccine to those from 50–60 years to 85 years. Serious cardiovascular complications including thrombosis and myocarditis have been reported with other COVID-19 vaccines too, including the two mRNA vaccines, the Pfizer-Biotech and the Moderna vaccine [12,13]. Vaccine-associated anaphylaxis, although rare, is another AESI reported with Covishield [3], Covaxin [4], and other COVID-19 vaccines [14,15]. We found one case of possible thrombosis/ thromboembolism due to Covishield and two cases of anaphylaxis (one each with Covishield and Covaxin) in our retrospective analysis.

We could analyze only three factors, that is, gender, the dose of vaccine (first or second dose), and category of the recipient (HCW or non-HCW) for risk of AEFI following the COVID-19 vaccine, since we only had information about these three factors in those who received a COVID-19 vaccination at the study center but did not report AEFI. We found no association between the risk of experiencing AEFI and gender with either vaccine. However, there were a significantly greater number of AEFI reported after the first dose of Covishield as well as Covaxin as compared to the second dose of either vaccine. The proportion of total and non-HCW vaccine recipients who received their second dose was higher than those who received their first dose at our study site, the difference being greater for Covaxin. However, in the case of HCWs, there were more first doses as compared to second doses. This trend can be explained by the fact that only HCWs received the vaccine at healthcare institutes at the beginning of the COVID-19 vaccination drive, while by the time second doses became available, non-HCWs who had received their first doses elsewhere, got second doses at the study site. Despite a higher proportion of first doses administered, significantly more AEFI were reported after the first dose of either vaccine. This may be due to the higher levels of anxiety in relation to the hitherto unknown adverse effects of new vaccines among both the vaccines as well as the HCWs, which may have been allayed by the time the second doses were given.

In addition, non-HCWs had a higher rate of AEFI after receiving both vaccines as compared to HCWs. We could find no explanation for this difference in the AEFI rate among HCWs and non-HCWs. While several observational studies have analyzed the AEFI in HCWs in India, we did not find a study that has compared rates of AEFI in HCWs with non-HCWs [16,17]. In a prospective observational study of 981 HCWs in a tertiary care COVID-dedicated hospital in southern India, 57% of vaccine recipients reported at least one or more adverse events after the first dose, while 14.1% of recipients reported adverse events after the second dose. The higher rate could be due to the active surveillance of AEFI as opposed to the passive surveillance in our study. There was no association of AEFI with sex and profession but a significant association of AEFI was found with age (*p* < 0.01), with a higher incidence of AEFI in HCWs > 50 years old [16]. Similarly, active surveillance for AEFI with Covaxin in central India reported an AEFI rate of 29.8% [17].

A passive surveillance of 1634 armed forces HCWs who received their first dose of Covishield reported an AEFI incidence proportion of 6.4% (95% CI: 5.3%, 7.7%), which again is nearly ten times the AEFI rate in our study. All AEFI were minor, the most common being fever (65, 3.98%) and myalgia (54, 3.30%) [18]. Previous COVID-19 infection (aRR ¼ 2.40, 95% CI: 1.48, 3.91) and female sex (aRR ¼ 2.24, 95% CI: 1.22, 4.09) were significant independent risk factors for any systemic AEFI reported after COVID-19 vaccination [17]. Previous COVID-19 infection and female sex, along with full vaccination dose and younger age, were associated with greater odds of adverse effects in a systematic review of adverse events with mRNA vaccines also [19]. Female sex and younger age were also associated with higher rates of AEFI due to Covishield in a cross-sectional online survey on AEFI in the general population in Vietnam [20], and with Covaxin in a study in central India [17].

Comparing AEFI due to Covishield with Covaxin, there were more vaccinees with a co-morbidity as compared to those with no co-morbidity in the Covaxin group; the ratio was reversed in the Covishield group with only 64 vaccinees having a co-morbidity, while the majority did not have any concurrent disease. Since we do not know the rate of co-morbidity in all vaccine recipients, this difference may be due to the different baseline characteristics of the vaccines.

There are a few limitations in this study. The study data are based on passive surveillance, that is, the stimulated spontaneous reporting of AEFI, and participants were not followed up actively. Many minor AEFI, for example, fever and pain at the injection site may have not been reported. Moreover, our data of Covishield and Covaxin recipients did not include recipient identity, therefore our denominator is the total number of either vaccine administered, not the actual number of men and women who have received the vaccine as one individual received two doses. What is more, the actual age of recipients was not available. As there were many changes in the COVID-19 vaccination policies and practices in the country during the study period, the data for the type of vaccine and the age population is not uniformly distributed. COVID-19 vaccination at the study site started on 11 January 2021 with the administration of Covishield to healthcare workers and other frontline workers during the pandemic, while Covaxin administration started on 26 April 2021. Since 1 March 2021, COVID-19 vaccination was made available to non-HCWs above the age of 65 years and above 45 years with comorbidities, and from 1 May 2021, it was made available to adults aged from 18 to 44 years as well. Due to these limitations, and the relatively small number of AEFI, the power to identify statistically significant differences in sub-groups was limited.

There are a few strengths to this study. The study has a large sample size with a long study duration. The study data includes the AEFI reported with the two most commonly administered vaccines in the Indian population and includes both HCWs and non-HCWs. The study also highlights the varied pattern of symptoms reported in the AEFI due to COVID-19 vaccination.

## 5. Conclusions

As compared to the Indian national data, higher overall rates of total AEFI and serious AEFI with COVID-19 vaccines were found in the study, which may be due to stimulated AEFI reporting. AEFI rates with Covishield and Covaxin were found to be similar. Total AEFI with the two COVID-19 vaccines were uncommon, and serious AEFI were rare. We also found a higher rate of AEFI in non-HCWs and those receiving their first dose of the COVID-19 vaccine. Whether immunological tolerance or allayed anxiety was responsible for lower AEFI risk with the second dose remains to be investigated.

## Figures and Tables

**Table 1:** Baseline characteristics and co-morbidity status of cases reporting AEFI.

Characteristic	Category	Covishield		Covaxin		Total	
		Frequency *n* (%) (*N* = 307)	% of Doses (*N* * = 41,462)	Frequency *n* (%) (*N* = 23)	% of Doses (*N* * = 9548)	Frequency (%) (*N* = 330)	% of COVID Vaccine Doses (*N* * = 51,010)
Gender	Male	176 (57.33)	0.42	12 (52.17)	0.13	188 (56.97)	0.37
	Female	131 (42.67)	0.32	11 (47.83)	0.12	142 (43.03)	0.28
Dose distribution	First dose	290 (94.46)	0.7	18 (78.26)	0.19	308 (93.33)	0.6
	Second dose	17 (5.54)	0.04	5 (21.74)	0.05	22 (6.67)	0.04
Designation	Physician	59 (19.22)	0.14	2 (8.7)	0.02	61 (18.48)	0.12
	Nurse	36 (11.73)	0.09	0	0	36 (10.91)	0.07
	Pharmacist	5 (1.63)	0.01	1 (4.35)	0.01	6 (1.82)	0.01
	Other HCW	24 (7.82)	0.06	1 (4.35)	0.01	25 (7.58)	0.05
	Non-HCW	183 (59.61)	0.44	19 (82.61)	0.20	202 (61.21)	0.4
History of medical disorder	Yes	248 (80.78)	0.6	20 (86.96)	0.21	268 (81.21)	0.53
	No	59 (19.22)	0.14	3 (13.04)	0.03	62 (18.79)	0.04
History of COVID-19 infection	Yes	301 (98.05)	0.73	23 (100)	0.24	324 (98.18)	0.64
	No	6 (1.95)	0.01	0	0.00	6 (1.82)	0.001
Total		307 (100)	0.74	23 (100)	0.24	330 (100)	0.65

*N* * indicates total number of vaccine doses administered at study center, not number of recipients since same patient may have been counted twice, once while receiving first dose, and again when receiving second dose.

**Table 2:** Characteristics of AEFI reported with COVID-19 vaccination.

Characteristic	Category	Covishield		Covaxin		Total	
		Frequency *n* (%) (*N* = 307)	% of Doses (*N* = 41,462)	Frequency *n* (%) (*N* = 23)	% of Doses (*N* = 9548)	Frequency (%) (*N* = 330)	% of COVID Vaccine Doses (*N* = 51,010)
Lag Period *	<1 h	17 (5.54)	0.04	3 (13.04)	0.03	20 (6.06)	0.04
	1–24 h	272 (88.6)	0.66	20 (86.96)	0.21	292 (88.48)	0.57
	2–7 (days)	10 (3.26)	0.02	0	0	10 (3.03)	0.02
	>7 (days)	8 (2.61)	0.02	0	0	8 (2.42)	0.02
Duration of AEFI ** (Days)	<1	12 (3.91)	0.03	2 (9.09)	0.02	14 (4.26)	0.03
	1–5	262 (85.34)	0.63	17 (77.27)	0.18	279 (84.8)	0.55
	6–10	22 (7.17)	0.05	2 (9.09)	0.02	24 (7.29)	0.05
	>10	11 (3.58)	0.03	1 (4.55)	0.01	12 (3.65)	0.02
Seriousness	Serious AEFI	4 (1.3)	0.01	2 (8.7)	0.02	6 (1.82)	0.01
	Non-serious AEFI	303 (98.7)	0.73	21 (91.3)	0.22	324 (98.18)	0.64
Causality	Certain	59 (16.3)	0.14	1 (4.17)	0.01	60 (15.54)	0.12
	Probable	291 (80.39)	0.7	22 (91.67)	0.23	313 (81.09)	0.61
	Possible	7 (1.93)	0.02	1 (4.17)	0.01	8 (2.07)	0.02
	Unlikely	5 (1.38)	0.01	0	0	5 (1.3)	0.01
Outcome	Recovered	307 (100)	0.74	22 (95.65)	0.23	329 (99.7)	0.64
	Recovering	0	0	1 (4.35)	0.01	1(0.3)	0.001
Treatment received for AEFI	Yes	282 (91.86)	0.68	22 (95.65)	0.23	304 (92.12)	0.6
	No	25 (8.14)	0.06	1 (4.35)	0.01	26 (7.88)	0.05
Total		307 (100)	0.74	23 (100)	0.24	330 (100)	0.65

* Maximum lag period-Covishield = 672 h (28 days)—Covaxin = 24 h. ** Maximum duration-Covishield = 24 days—Covaxin = 11 days.

**Table 3:** System-wise distribution of AEFI reported with COVID-19 vaccination.

Characteristic	Covishield		Covaxin		Total	
	Frequency *n* (%) of AEFI Symptoms (*N* = 844) *	% of Doses (*N* = 41,462)	Frequency *n* (%) of AEFI Symptoms (*N* = 56) *	% of doses (*n* = 9548)	Frequency *n* (%) of AEFI Symptoms (*N* = 900) *	% of COVID Vaccine Doses (*N* = 51,010)
Generalized symptoms	409 (48.46)	0.99	19 (33.93)	0.2	428 (47.56)	0.84
Pain **	249 (29.5)	0.07	13 (23.21)	0.14	262 (29.11)	0.51
Gastrointestinal	55 (6.52)	0.13	3 (5.36)	0.03	58 (6.44)	0.11
Central Nervous System	40 (4.74)	0.1	5 (8.93)	0.05	45 (5)	0.09
Cardiovascular	20 (2.37)	0.05	7 (12.5)	0.07	27 (3)	0.05
Respiratory	30 (3.55)	0.07	1 (1.79)	0.01	31 (3.44)	0.06
Ophthalmological	8 (0.95)	0.02	2 (3.57)	0.02	10 (1.11)	0.02
Cutaneous reactions	7 (0.83)	0.02	3 (5.36)	0.03	10 (1.11)	0.02
Anaphylaxis	1 (0.12)	0	1 (1.79)	0.01	2 (0.22)	0
Other	25 (2.96)	0.06	2 (3.57)	0.02	27 (3)	0.05
Total	844 (100)	2.04	56 (100)	0.59	900 (100)	1.76

* Many patients had more than one AEFI symptom. ** Includes body aches, headaches, back pain, and limb/joint pain.

**Table 4:** Most frequent systemic AEFI with COVID-19 Vaccines.

Characteristic	Category	Covishield		Covaxin		Total	
		Frequency *n* (%) of AEFI Symptoms (*N* = 844) *	% of Covishield Doses (*N* = 41462)	Frequency *n* (%) of AEFI Symptoms (*N* = 56) *	% of Covaxin Doses (*N* = 9548)	Frequency *n* (%) of Total AEFI Symptoms (*N* = 900) *	% of COVID Vaccine Doses (*N* = 51,010)
Generalized symptoms	Fever	233 (27.61)	0.56	11 (19.64)	0.12	244 (27.11)	0.48
	Chills	75 (8.89)	0.18	2 (3.57)	0.02	77 (8.56)	0.15
	Weakness	33 (3.91)	0.08	2 (3.57)	0.02	35 (3.89)	0.07
	Shivering/Rigors	26 (3.08)	0.06	0	0	26 (2.89)	0.05
	Malaise	22 (2.61)	0.05	0	0	22 (2.44)	0.04
Pain	Body ache /Body Stiffness/Myalgia	122 (14.45)	0.29	6 (10.71)	0.06	128 (14.22)	0.25
	Headache	105 (12.44)	0.25	6 (10.71)	0.06	111 (12.33)	0.22
	Joint/limb pain/stiffness	14 (1.66)	0.03	1 (1.79)	0.01	15 (1.67)	0.03
Gastrointestinal symptoms	Nausea	28 (3.32)	0.07	1 (1.79)	0.01	29 (3.22)	0.06
	Vomiting	17 (2.01)	0.04	0	0	17 (1.89)	0.03
Central Nervous System symptoms	Dizziness	24 (2.84)	0.06	4 (7.14)	0.04	28 (3.11)	0.05
Respiratory symptoms	Cough	11 (1.3)	0.03	1 (1.79)	0.01	12 (1.33)	0.02
Cardiovascular symptoms	Hypertension	6 (0.71)	0.01	1 (1.79)	0.01	7 (0.78)	0.01
	Chest Discomfort/Chest Pain	5 (0.59)	0.01	2 (3.57)	0.02	7 (0.78)	0.01
Total		721 (85.43)	1.74	37 (66.07)	0.39	758 (84.22)	1.49

* Many patients had more than one AEFI symptom.

**Table 5:** Comparison between AEFI due to Covishield and Covaxin.

Risk Factors	Frequency *n* (%) of AEFI Reported due to Covishield (*N* = 41,462)	Frequency *n* (%) of AEFI Reported due to Covaxin (*N* = 9548)	*p*-Value
Male	176 (0.42)	12 (0.13)	0.63
Female	131 (0.32)	11 (0.12)	
Dose 1	290 (0.70)	18 (0.19)	0.003
Dose 2	17 (0.04)	5 (0.05)	
HCWs	124 (0.30)	4 (0.04)	0.029
Non-HCWs	183 (0.44)	19 (0.20)	
Serious	4 (0.01)	2 (0.02)	0.010
Non-serious	303 (0.73)	21 (0.22)	
Duration of AEFI ≤ 5 days	274 (0.66)	19 (0.20)	0.675
Duration of AEFI > 5 days	33 (0.08)	3 (0.03)	
Causality Certain/Probable	350 (0.84)	23 (0.24)	0.822
Causality Possible/Unlikely	12 (0.03)	1 (0.01)	
Treatment received for AEFI	282 (0.68)	22 (0.23)	0.514
No treatment received	25 (0.06)	1 (0.01)	
Any medical co-morbidity	64 (0.15)	20 (0.21)	<0.0001
No co-morbidity	248 (0.60)	5 (0.05)	
Had history of COVID-19 infection	6 (0.01)	0 (0.00)	-
No history of COVID-19 infection	301 (0.73)	23 (0.24)	
Recovering	0 (0.00)	1 (0.01)	-
Recovered	307 (0.74)	22 (0.23)	
Lag Period ≤1 (hour)	17 (0.04)	3 (0.03)	0.145
Lag Period >1 (hour)	290 (0.70)	20 (0.21)	
Lag Period ≤24 (hour)	289 (0.70)	23 (0.24)	-
Lag Period >24 (hour)	18 (0.04)	0 (0.00)	

## Data Availability

The study site is a designated Regional Training Centre for Pharmacovigilance cum Adverse Drug Monitoring Centre under the Pharmacovigilance Programme of India. All Adverse Drug Reaction reports received at the centre are sent via Vigiflow to the Indian Pharmacopeia Commission, Ghaziabad, which is the National Coordinating Centre for the Pharmacovigilance Programme of India. Data used in the study has been collected as part of the routine functioning of the Centre.
